# Explaining Sex Differences in Motorcyclist Riding Behavior: An Application of Multi-Group Structural Equation Modeling

**DOI:** 10.3390/ijerph17238797

**Published:** 2020-11-26

**Authors:** Savalee Uttra, Napat Laddawan, Vatanavongs Ratanavaraha, Sajjakaj Jomnonkwao

**Affiliations:** School of Transportation Engineering, Institute of Engineering, Suranaree University of Technology, Nakhon Ratchasima 30000, Thailand; savalee.utt@gmail.com (S.U.); napat.l@g.sut.ac.th (N.L.); vatanavongs@g.sut.ac.th (V.R.)

**Keywords:** theory of planned behavior (TPB), locus of control (LC), multi-group SEM, MRBQ

## Abstract

Road accidents are caused by humans, vehicles, and road environments. Human attitudes affect behavioral changes and can lead to unsafe riding behavior. The sex of an individual is a key factor that affects their riding behavior. We aimed to use structural equation modeling (SEM) by analyzing the multi-group SEM between men and women and applying the theory of planned behavior (TPB) and the locus of control (LC) theory. The data used in the research were collected from all over Thailand, consisting of 1516 motorcycle riders (903 men and 613 women) aged over 20 years. A self-administered questionnaire was designed for data collection of the riding behavior using the Motorcycle Rider Behavior Questionnaire (MRBQ), including traffic errors, control errors, stunt frequency, and safety equipment. We found that riding behaviors between men and women were significantly different in both theories. For men, TPB showed that the main factors that highly influenced motorcycle riding behavior (MRB) were the attitudes based on health motivation (AHM) and perceived behavior control (PC); for women, AHM produced a stronger effect than in men. However, for the subjective norms (SN) factor, we found no direct effect on MRB, but did find an indirect effect through the attitudes based on severity (ASE) in both sexes. Particularly for women, the indirect influence value of the SN factor was higher. For women, the LC showed that internal factors had more influence than external factors. The same was found for men, but the effect in women was significantly stronger. We found that sex significantly affected the MRB. Therefore, policies must be implemented that address each group specifically as their attitudes and behaviors are different.

## 1. Introduction

### 1.1. Background

Vehicular collisions damage property and can be harmful to the health, even causing death. The number of motor vehicle collisions in Thailand remains high. According to road collision reports from the Royal Thai Police in 2019, 99,087 incidents were confirmed, of which 36,797 involved motorcycles. Of these, 65.5% were caused by humans, while traffic signals/traffic signs, vehicles, and the environment were involved in 27.5%, 5.1%, and 1.9%, respectively [1]. These incidents resulted in 8648 deaths (6473 men and 2175 women) [1]. Accidents involving serious or minor injuries totaled 61,101 (39,231 men and 21,870 women) [1]. These incidents also caused vast damages [2], estimated at 64.8 million Baht [1].

These collisions were caused by human, vehicle, and road environment factors. Humans are the key factor leading to collisions [3,4]. Evans [5] and Shinar [4] specified that human factors are a major cause of 95% of collisions, whereas road and vehicle factors are major causes of 28% and 8% of collisions, respectively. To understand human behavior from the perspective of vehicle collisions, Olson and Dewar [3] specified relevant human factors that cause accidents: rider perception and response when riding, individual differences, emotion, pressure, aggression, motivation, riding skill, risk behavior, social variables, rider attitude, rider sex, riding experience, fatigue, alcohol consumption, drunk riding behavior, age, and other physical characteristics. The human factor is complex due to people differing in terms of their physical and behavioral characteristics, such as their sex, age, interests, and motivations for riding behavior, which create different risks. To understand the behavioral aspect and solve problems regarding the human factors, individual attitudes must be studied to help create guidelines.

Demographics (sex and age) are considered as a basic variable of analysis. We wanted to study the same factors that affect motorcycle riding accidents as those considered in previous studies, such as Elliott and Thomson [6]. Useche, et al. [7] considered sex to study the difference of in risky bicycles cycling behavior using multi-group structural equation modeling (SEM). They found that both men and women have differences in terms of the hourly intensity of riding, psychological distress, and the level of knowledge of traffic rules. In regard to the hourly intensity of riding, women reported higher intensities than men. For psychological distress, they found that men reported higher risky behavior compared with women. The knowledge of traffic rules was better for men than for women.

In terms of age and risk perception, they found significant results only in men. When considering positive behaviors, they found that age and psychological distress had no effects on men, and that age only affected women. In conclusion, sex differences can be supported in predicting the cycling behavior of male and female bicyclists [7]. Martinussen et al. [8] constructed a car driving behavior measurement model using the driver behavior questionnaire (DBQ) [9]. The analysis also created groups structured by age, sex, and driving distance using exploratory and confirmatory factor analysis.

The statistics of accidental deaths classified by gender in Thailand, as reported by the Royal Thai Police, found that more males died from accidents than females. In addition, in many past research studies based on sex differences in driving behavior, the majority found differences. Among human factors, the sex of an individual is a key factor that affects their riding behaviors, as has been stated in past studies. We focused on sex differences as the main point of this research in order to test and verify that the differences between males and females contribute to different driving behaviors. Therefore, policies must be implemented that address each group specifically as their attitudes and behaviors are different.

### 1.2. Literature Review

Various theories related to motivation in motor vehicle driving behavior have been widely used, such as the health belief model (HBM) [10], the theory of planned behavior (TPB) [11], and locus of control (LC) [12]. These theories are very useful in studying motivation for motorcycling behavior based on sex differences. Motorcycle driving behavior is a behavior that drivers regularly practice and become habituated to and, thus, have motivation or attitudes regarding the behavior. Therefore, the theory of TPB and LC is suitable for this study of behavior.

#### 1.2.1. Theory of Planned Behavior (TPB)

Ajzen [11] explained the TPB as the study of the influence of attitude on behavioral change. This theory is a result of developments from the theory of reasoned action, which is social psychology developed from the theory of reasoned action by Bamberg, et al. [13]. TPB explains that human behavioral expression is generated from three kinds of belief: behavioral, normative, and control beliefs; each belief affects different variables [11].

Attitudes toward behavior enable individual assessments of behaviors that are created by relevant beliefs toward behavioral expression (behavioral beliefs), as well as assessments or judgments of negative or positive behavioral results. If an individual assessment of a result is positive, then this individual will have a positive attitude toward the behavior that they observe, and vice versa. A subjective norm behavior is an individual perception of a social demand for a person to conduct or not conduct a certain behavior. Subjective norm behavior is generated by individual beliefs toward social demand normative actions, particularly when these actions are conducted by others that are important to that individual, e.g., family members, close friends, and partners.

Xiao [14] used TPB and SEM to study vehicle driving behavior and found that perceived behavioral control can directly and indirectly predict self-reported unsafe driving behavior. Razmara, et al. [15] used multiple regression analysis to determine that subjective norms, perceived behavioral control, and habits were the main predictors of one’s intention to drive safely. Bazargan-Hejazi, et al. [16] used multiple regression analysis to find that attitude was the strongest predictor of intention. Intention was found to mediate the relationship between willingness to text while driving (TWD) and perceived behavioral control. Li, et al. [17] used confirmatory factor analysis (CFA) and structural equation modeling (SEM) techniques and found that TPB was an accurate predictor of competitive behavior intention, and a high correlation between the dimensions of social environment and intention was observed. Differences in driver competition were also observed between sexes.

#### 1.2.2. Locus of Control (LC)

The concept of Locus of Control (LC) was developed by Rotter [12], rooted in the concept of social learning behavior theory, consisting of four main aspects: (1) behavior potential, (2) expectancy, (3) reinforcement value, and (4) psychological situation. Control factors are divided into an internal and external locus of control. The internal locus of control can be explained by a personal attitude which accepts that the consequences of an action were subject, and can be controlled by themselves. Whereas, the outside of control was the external locus of control [12].

Therefore, Montag and Comrey [18] applied LC to studied on driving, they separated Driving Internality (DI) and Driving Externality (DE). The result from found that DI and DE had stronger relationship with safety driving. Then, Arthur and Doverspike [19] studied DI had effect to accidents rates more than DE.

In addition, LC for measuring driver locus of control, risky driving and negative outcomes was developed by Özkan and Lajunen [20]. They developed Multidimensional Traffic Locus of Control Scale (T-LOC) for drivers including: “Other Drivers”, “Self”, “Fate”, and “Vehicle and Environment”. In addition, LC and T-LOC have been developed, such as Swedish driver version [21], and Romanian driver version [22].

Măirean et al., 2017 [22] had suggested the relation between T-LOC and driving behavior is not always clear and the evidence on the relation between T-LOC, risk perception, and risky behavior is somewhat mixed. Therefore, the question about the role of LC in risky driving behavior is still open.

Moreover, LC was studied by Champahom, et al. [23], they studied vehicle riding behavior by considering helmet wearing behavior among downtown and suburban residents. Lajunen and Räsänen [24] used the LC to examine bicycle using behavior. Totkova [25] analyzed individual riding patterns activated by anger, anxiety, as well as dissociative, distress-reduction, high velocity, irrational, patient, careful, and risky styles.

#### 1.2.3. Motorcycle Rider Behavior Questionnaire (MRBQ)

The MRBQ was developed by [26] from the driver behavior questionnaire (DBQ) by Reason et al. [9]. Elliott et al. [26] studied factors influencing rider behavior using principal component analysis (PCA) to divide forms of the factors, including traffic errors, control errors, speed violations, performance of stunts, and the use of safety equipment. The MRBQ has 43 questions that were used for linear modeling with age, experience, and riding distance/years. Rider behaviors were measured using five measurement factors, namely, traffic errors, control errors, speed violations, stunts, and safety equipment.

Traffic errors referred to a factor instead of making mistakes or making wrong decisions while driving. “Control errors” referred to variables of error handling behavior (slipping). “Speed violations” referred to the behavior variables involved in speed violations. “Stunts” referred to the main variables of behavior that are involved in thrilling and extreme driving. Finally, “Safety equipment” referred to the use of equipment variables that improve driving safety [26].

The MRBQ has been widely used in studies of motorcycle riding behavior, such as Özkan, et al. [27]. Uttra, et al. [28] developed the MRBQ as an assessment tool for the riding behavior of Thai people, consisting of 26 questions for four factors: traffic errors, control errors, stunts, and safety equipment. We selected these four factors to further study the motivating theory of behavioral practice in structural equation modeling.

#### 1.2.4. Structural Equation Modeling (SEM)

We were interested in theories concerning the motivation or attitudes that resulted in a behavior with several related variables and factors. Therefore, structural equation modeling (SEM) was the most appropriate statistical equation method for this study.

Khampirat [29], in reference to Ratanavaraha, et al. [30], reported that SEM, which is also called covariance structure analysis or the LISREL model, is a model that expresses relationships between latent variables, as well as between latent variables and indicators. SEM is a result of the synthesis of important data analysis from three methods: factor, path, and parameter estimation regression analyses. The SEM consists of two sub-models: the measurement model and structural model.

Nguyen, et al. [31] applied SEM for the analysis of motorcyclists’ cellphone attention behavior based on the theory of planned behavior (TPB) in Vietnam, and the results from the SEM analysis showed good fits to the observed data. The SEM analysis also supported the utilization of an extended TPB framework in identifying the factors of mobile phone use with riding intention and behavior.

### 1.3. Objective, Hypothesis, and Contributions of this Research

Collisions are often due to human behaviors and attitudes, and the influence of attitude on behavioral changes can generate unsafe riding behavior. We used psychological concepts and theories relevant to behavioral influence, TPB, and LC to predict accidents so we could identify the factors that affect accident occurrence. This also involved using multiple regression analysis, CFA, and SEM. The results can be used to provide guidelines for road safety policies. No research has previously been conducted with SEM along with TPB and LC theories that divides participants by their sex, which is a key factor of individual riding behavior. Therefore, we also aimed to construct a structural equation model by analyzing multi-group SEM between men and women so we could then analyze the variance of participants by applying TPB and LC. Our hypotheses were constructed to seek answers to these questions and fill the aforementioned gap in the literature:

**Hypothesis** **1** **(H1).**  *There is no difference in the invariance between sexes as determined by the TPB.*

**Hypothesis** **2** **(H2).**  *There is no difference in the variance between sexes as determined by the LC.*

**Hypothesis** **3** **(H3).**  *Perceived behavioral control has a negative effect on rider behavior (MRBQ).*

**Hypothesis** **4** **(H4).**  *Health motivation attitude has a negative effect on rider behavior (MRBQ).*

**Hypothesis** **5** **(H5).**  *Attitude (severity) has a negative effect on rider behavior (MRBQ).*

**Hypothesis** **6** **(H6).**  *Subjective norm has a negative effect on rider behavior (MRBQ).*

**Hypothesis** **7** **(H7).**  *An external locus of control negatively affects rider behavior (MRBQ).*

**Hypothesis** **8** **(H8).**  *An internal locus of control negatively affects rider behavior (MRBQ).*

The contribution of this study was to focus on the factors influencing vehicle driving behavior at a significant level in both negative and positive signals. Particularly, the positive influence results could be used to provide guidelines for road safety policies, whereas the negative influence could be an aid in the campaign of Reducing/Abandoning/Quitting, to enhance the strategy of promoting public relations campaigns in directions consistent with different contexts.

The remainder of this paper is structured as follows: In Section 2, we describe the methods, including the participants, measures, instruments, and data analysis. In Section 3, we outline the results, including the descriptive statistics, results of factor analysis, reliability, multi-group SEM, and the model estimate. The discussion and conclusions are described in Section 4, and the limitations and future work are described in Section 5.

## 2. Method

### 2.1. Participants

The participants were gathered from all over Thailand distributed to five administrative regions, consisting of Bangkok and its vicinities, the central region, the northeast, the north, and the south. The respondents were required to be over 20 years of age, able to ride motorcycles, have experience in motorcycling, and with or without driving licenses. The total samples in this research (1516) were deemed sufficient for the analysis of structural equation models as Golob [32] stated that n should be equal to 15 times the indicator factors [33].

The sample profile (Table 1) included 903 men (59.6%) and 613 women (40.4%), with average ages of 36.4 and 33.2 years, respectively. The most common education level was a bachelor’s degree (47.40% of men and 54.81% of women). In terms of career, most of the male participants owned a private company, followed by general worker as an occupation at 40.20% and 21.37%, respectively. Female participants also most commonly owned private companies, followed by personal business/trading owner at 46.82% and 17.29%, respectively. We found that both the male and female participants commonly had motorcycle riding licenses (94.91% and 86.13%, respectively).

### 2.2. Measures and Instrument

The MRBQ was developed by Elliott et al. [26] as an assessment tool of the frequency of motorcycle rider behavior from the DBQ [9]. Elliott et al. [26] constructed 46 MRBQ indicators that allowing participants to complete a self-assessment, choosing one answer per question. The answers were provided on a 6-point scale (1 = never, 2 = sometimes, 3 = often, 4 = always, 5 = nearly all the time, and 6 = all the time). The five factors that measured motorcycle riding behavior were traffic errors, speed violations, stunt frequency, safety equipment, and control errors. In this study, MRBQ was specified as a dependent variable in the SEM.

Independent variable was developed to collect data regarding the attitudes that affect the MRBQ by reviewing the TPB and LC. The TPB has been developed to cover attitudes toward behavior, subjective norm, and perceived behavior control. The LC and T-LOC have been used in recent driving studies which are useful for further study and application to the context of motorcycling behavior of Thai people. Moreover, most of the factors associated with accidents are known to be caused by human factors [3,4,5] and behavioral approaches [11,34]. Therefore, in order to continue the development, the LC can be implemented and easy to understand. This research has developed LC indicators affecting MRBQ rider in the Thai context. The attitude that comes from Internal factor (My own decision) and External factors derived from environment related attitude (other people [35]: family and friends [36,37], accident situation or accident news, road safety campaign [38], and strictly police in traffic law [39]), which may contribute to safer driving behaviors used in this study.

The questionnaire was designed as a self-assessment, choosing one answer per question. The questions were answered on a seven-point scale in accordance with level of behavioral agreement (1 = do not agree to 7 = strongly agree). An example of a statement used in the questionnaire is: “If you get into an accident, your health and physical body will not be the same.”

These research tools were adjusted to suit Thai people’s behavior. They were tested prior to data collection using the objective congruence index (IOC) with seven measurements developed by traffic and transport, safety, and education experts who had the knowledge needed to be able to assess the research tool. Then, the experiment was run by collecting 100 sets of data and testing the data’s normal distribution [40] and calculating Cronbach’s α, which was required to be higher than or equal to 0.7 [41]. This research was approved by the Ethics Committee for Research Involving Human Subjects, Suranaree University of Technology (Pr: EC-63-0052).

### 2.3. Data Analysis

#### 2.3.1. Factor Analysis

Factor analysis was used to study the measurements of observed variables or indicators, and is a distribution technique for observed variables or indicators that can be directly measured [42] in the simple forms of exploratory factor analysis (EFA) and confirmatory factor analysis (CFA).

SPSS 18.0 software (SPSS Inc., Chicago, IL, USA) was used in the analysis of EFA, and factor analysis was used to classify or decrease the numbers of variables in the observed variables or indicators [43]. Factor loadings > 0.5 were considered for further study [9]. This research consisted of 20 observed variables that were analyzed under the TPB and LC criteria.

#### 2.3.2. Multi-Group SEM

Here, with multi-group SEM analysis, we focused on attitudes according to TPB and LC that affect motorcycle rider behavior (MRB) and the differences between men and women. We used SEM and multi-group analysis to test the hypotheses using Mplus 7.2 software [44].

For hypotheses testing, the following criteria were used: goodness-of-fit-statistics with chi-squared/degree of freedom (df) < 5 [45], root mean squared error of approximation (RMSEA) < 0.08 [46,47], standardized root mean square residual (SRMR) < 0.08 [45], comparative fit index (CFI) ≥ 0.90 [45], and the Tucker–Lewis index (TLI) ≥ 0.80 [48,49].

## 3. Results

### 3.1. Descriptive Statistics

The calculated descriptive statistics (mean, standard deviation (SD), skewness, and kurtosis; Table 2) showed that men could be classified into four latent variables (Traffic Error (TE), Control Error (CE), Stunt (ST), and Safety Equipment (SE)) for the MRBQ variables, and the mean for men was between 1.28 and 2.33. The mean for women was between 1.25 and 2.21. The SD was 0.51–0.88 and 0.48–0.79 for men and women, respectively. The skewness was between −0.805 to 1.490 in men and −0.740 to 2.081 in women. The kurtosis was −1.250 to 1.217 and −1.303 to 3.575 for men and women, respectively. From the analysis results, we concluded that the MRBQ kurtosis and skewness values were less than 3 and 10 for men and women, respectively [40].

Among the variable groups of the TPB and LC (X1–X20), we found that the means for men and women were 4.68–6.60 and 4.98–6.62, respectively. The SD in men was 0.63–1.18 and was 0.60–1.31 for women. The skewness was between −1.581 and 0.303 in men and −1.588 and 0.136 in women. The kurtosis was between −0.700 and 2.268 in men and −0.908 and 2.512 in women. We found that these results passed, which states that skewness should be less than 3.0 and kurtosis should be less than 10.0.

### 3.2. Factor Analysis Results

The factor analysis results dividing men and women (Table 3) was the outcome of the EFA of men according to TPB. We found four factors of EFA, including attitudes based on health motivation (AHM), attitudes based on severity (ASE), subjective norm (SN), and perceived behavior control (PC). EFA, in accordance with LC theory, identified two factors: externality (EX) and internality (IN). The EFA used PCA as the extraction method and Varimax with Kaiser normalization as the rotation method. The Kaiser–Meyer–Olkin (KMO) was 0.774, and the EFA’s factor loadings of TPB and LC theory were 0.664–0.900 and 0.746–0.880, respectively. Table 4 provides the results for women; the factor loadings using EFA from TPB and LC theory were 0.736–0.900 and 0.734–0.901, respectively.

CFA was performed before SEM to confirm both the indicators and factors. In men, the factor loading of TPB was between 0.510 and 0.981 and of LC was between 0.598 and 0.962. The CFA of MRBQ showed that the factor loading was between 0.423 and 0.843 for TPB analysis and 0.449 and 0.884 for LC analysis.

Table 4 provides the results of the EFA and CFA for female riders, with a KMO of 0.791 for TPB and 0.754 for LC. Factor loading obtained by EFA was between 0.549 and 0.866 for TPB and was between 0.542 and 0.813 for LC theory. CFA was also performed, which showed that the factor loading was between 0.489 and 0.979 for TPB and 0.579 and 0.948 for LC.

The CFA obtained by MRBQ analysis found that factor loading in TPB was 0.360–0.817. For LC, the factor loading was 0.300–0.945.

### 3.3. Reliability

The accuracy of indicators was indicated by Cronbach’s *α* values of 0.7 or higher [41]. The TPB analysis consisted of four variables: AHM, ASE, SN, and PC. In men, the Cronbach’s *α* values were 0.806, 0.927, 0.856, and 0.864, respectively. LC theory analysis produced two variables: EX and IN. For men, the Cronbach’s *α* values were 0.889 and 0.864, respectively (Table 3). For women (Table 4), the Cronbach’s *α* values were 0.805–0.932 for the TPB analysis and 0.842–0.921 for the LC analysis.

The composite reliability (CR) and average variance extracted (AVE) were respectively calculated using Equations (1) and (2):

(1) $$CR=\;\frac{{\left(\sum_{i=1}^{n}L_{i}\right)}^{2}}{{\left(\sum_{i=1}^{n}L_{i}\right)}^{2}+\left(\sum_{i=1}^{n}e_{i}\right)}$$

(2) $$AVE=\;\frac{\sum_{i=1}^{n}L_{i}}{n}$$

where *L_i_* is the standardized factor loadings obtained by CFA, *i* is the number of observed variables in each variable factor, and *e_i_* is the error variance terms of each group of measurement models under the condition *CR* ≥ 0.7 [42]. The *CR* was 0.812–0.930 for TPB and 0.859–0.913 for LC analysis with *AVE* ≥ 0.5 [42]. The analysis of men was 0.678–0.827 for TPB and 0.768–0.818 for LC (Table 4). For women (Table 4), the *CR* was 0.814–0.940 with TPB and 0.936–0.843 for LC. The AVE was between 0.678–0.852 for TPB and 0.860 and 0.751 for LC.

We tested for correlation to investigate the relationships between MRBQ (TE, CE, ST, and SE), TPB (AHM, ASE, SN, and PC), and LC (EX, IN) using a Pearson table at the 99% significance level. We found that men were between −0.479 and −0.066 and women were between −0.550 and −0.099 (Table 5).

### 3.4. Multi-Group Analysis

Multi-group SEM was used to test the invariance in the between-group model for men and women (Hypotheses 1 and 2) using TPB and LC theory. According to the results in Table 6, we found that the TPB measurements of invariance with factor loading hypothesis, intercepts, and structural paths were equal between the groups (model 3; Chi-square = 1420.039, df = 330, Chi-square/df = 4.69 (<5) [45], RMSEA = 0.070 (0.066–0.073) [46,47], CFI = 0.954 [45], TLI = 0.924 [48,49], and SRMR = 0.068 < 0.08 [45]). For model 4, the factor loading, intercept, and structural path were equal between the groups (Chi-square = 1529.940, df = 336, Chi-square/df = 4.55, RMSEA = 0.068 (0.065–0.072), CFI = 0.951, TLI = 0.926, and SRMR = 0.077). The analysis result of both models (models 3 and 4) showed the goodness of fit and met the criteria in accordance with specified values. The testing result of the difference between model 3 and model 4 produced a Chi-square of 109.901 with df = 33 at *p* < 0.01. In conclusion, we rejected the hypothesis 1 that the TPB’s SEM of motorcycle riding behaviors of men and women are different.

The LC measurement invariance (model 7) had a Chi-square of 213.265, a df of 67, and a Chi-square/df of 3.18, which is <5 [45] (RMSEA = 0.054 (0.046–0.062) [46,47], CFI = 0.987 [45], TLI = 0.970 [48,49], and SRMR = 0.042 < 0.08) [45]. Model 8 had a Chi-square of 277.877, a df of 84, and a Chi-square/df of 3.31 (RMSEA = 0.055 (0.048–0.062), CFI = 0.983, TLI = 0.968, and SRMR = 0.056). The analysis results of both models (models 7 and 8) had good fits and met the criteria. The testing result of the difference between the models was Chi-square = 64.612 with df = 17 at *p* < 0.01. In conclusion, we rejected the hypothesis that the LC’s structural equation model of motorcycle riding behavior of the samples of men and women are different.

### 3.5. Model Estimate

The analysis results of the SEMs for TPB and LC, which affect the MRB, could explain and express the factor loading of each indicator, as shown in Table 7 and Table 8.

#### 3.5.1. TPB Model Estimate for Men

**Figure 1:**
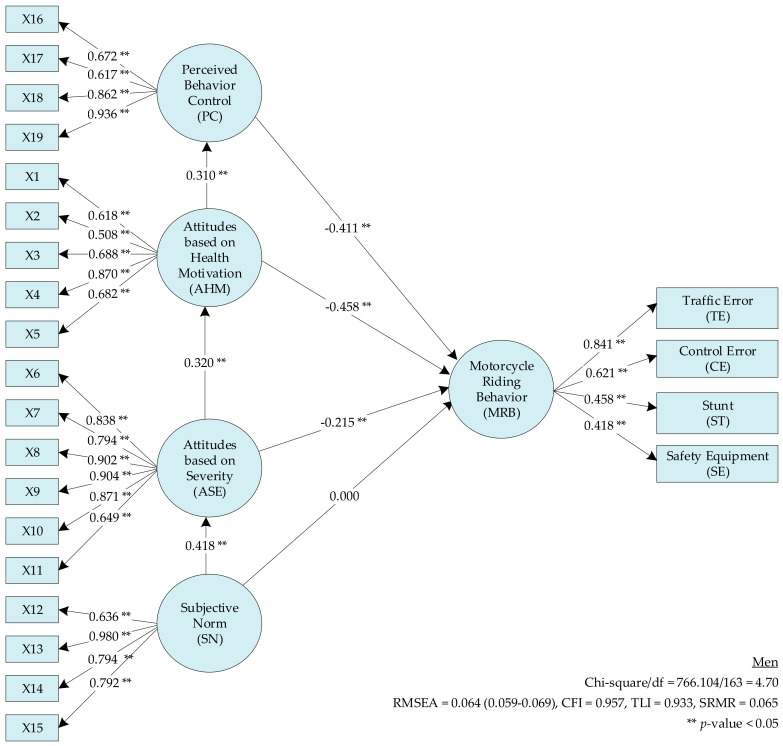
The theory of planned behavior model for men.

The SEM for the TPB (Figure 1) in men showed that H3 (perceived behavior control has a negative effect on rider behavior) was supported (β = −0.411, *p* < 0.05). H4 (attitude (health motivation) has a negative effect on rider behavior) was also supported (β = −0.458, *p* < 0.05). H4 and H5 (attitude (health motivation) and attitude (severity) have a negative effect on rider behavior, respectively) were supported (β = −0.458 and −0.215, respectively; *p* < 0.05). H6 (subjective norm) had no effect on the MRB. AHM, ASE, and SN were found to indirectly affect the MRB, as presented in Figure 1 and Table 9.

#### 3.5.2. TPB Model Estimate for Women

**Figure 2:**
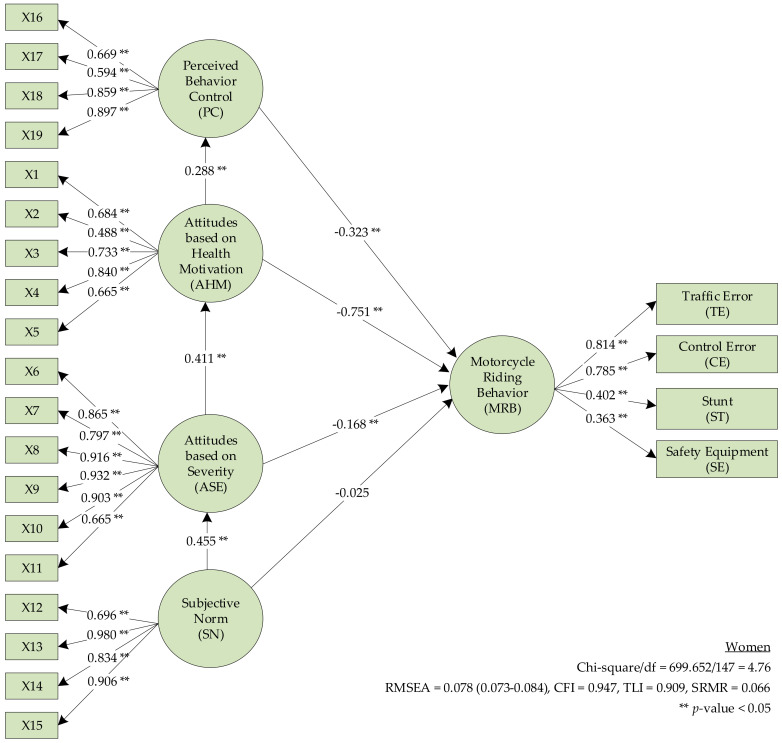
The theory of planned behavior model for women.

The SEM for TPB (Figure 1) in women showed that H3–H5 were supported (β = −0.323, −0.751, −0.168, respectively; *p* < 0.05). H6 (subjective norm) had a negative effect on MRB. AHM indirectly affected the MRB through PC, ASE indirectly affected the MRB through AHM, and SN had no direct effect on the MRB but indirectly had an effect through ASE as shown in Figure 2 and Table 9.

#### 3.5.3. LC Model Estimate for Men

**Figure 3:**
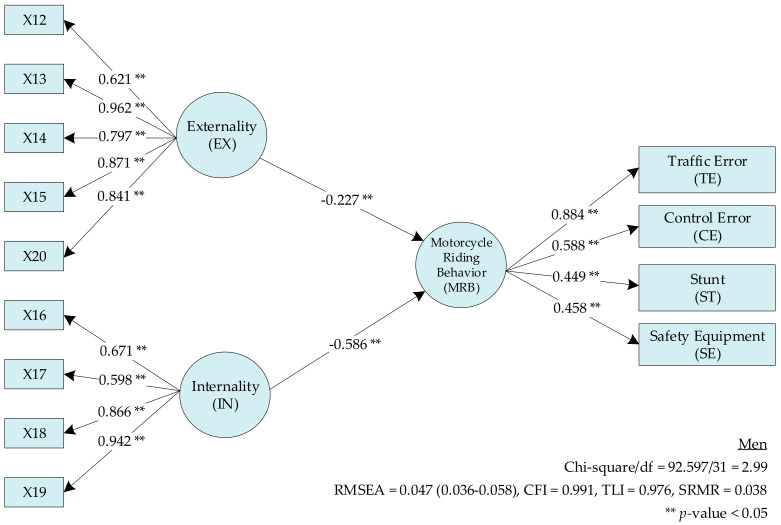
The locus of control model for men.

The SEM for the LC (Figure 3) in men showed that H7 (internality (IN)) most affected the MRB (β = −0.586; *p* < 0.05). H8 (externality (EX)) had a β of −0.227 (*p* < 0.05); thus, H7 and H8 were supported. The analysis results are presented in Table 8 and Table 9.

#### 3.5.4. LC Model Estimate for Women

**Figure 4:**
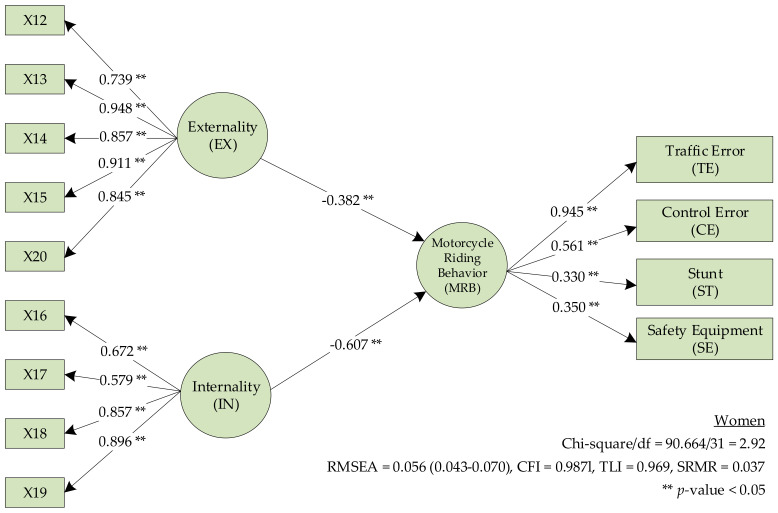
The locus of control model for women.

The SEM for the LC (Figure 4) in women found that H7 (IN affects the MRB) had a β value of −0.607. H8 (EX affects the MRB) had a β of −0.382 (*p* < 0.05); thus, H7 and H8 were supported. The analysis results are presented in Table 8 and Table 9.

## 4. Discussion and Conclusions

In this research, we developed a structural equation model by analyzing multi-group SEM between men and women through applying the theory of planned behavior (TPB) [11] and the locus of control (LC) theory [12]. From the developed SEM, we found that the leading attitudes of men and women toward riding behavior from both theories were significantly different, which is a finding in accordance those reported by Useche et al. [7], who studied sex differences in risky bicycle riding behavior. Martinussen et al. [8] found that the criteria for a good fit between men and women were significantly different. Our findings also agreed with those of Li et al. [17], who observed sex differences in driver competition.

### 4.1. Discussion of the TPB

Using multi-group SEM analysis with TPB, Ajzen [11] reported that the TPB led to the actual behavior. We found that in men, the attitude (health motivation) factor most strongly affected the riding behavior. This finding is supported by Bazargan-Hejazi et al. [16] and Nguyen et al. [31], who found that attitude had the greatest effect on riding behavior. The next most influential factor was perceived behavior control, in agreement with Xiao [14], Razmara et al. [15] and Bazargan-Hejazi et al. [16], who both reported the direct and indirect effects of this factor on the theory of planned behavior (TPB). Although we only found a direct influence, attitude (severity) was also found to influence riding as was also reported by Bazargan-Hejazi et al. [16].

For the women’s study results, we found that the attitude (health motivation) factor most affected the riding behavior, as reported by Bazargan-Hejazi et al. [11,16]. In comparison to men, we found that this factor received almost double the loading. The perceived behavior control factor finding also agrees with Razmara et al. [15] and Xiao [14]. Attitude (severity) also influenced riding as reported by Bazargan-Hejazi et al. [16].

A difference was found in the subjective norm (SN). Both Thai men and women showed no direct influence on the attitude toward behavior, which contrasts the findings of Razmara et al. [15]. However, we found that SN also indirectly affected the MRB through ASE, AHM, and PC.

The research results obtained by applying the TPB can provide a guideline for policy construction on road safety. Through SN analysis, we found no direct influence of this factor on either men or women. Therefore, encouraging riders to imitate the desired behavior or to follow referral groups or family may not affect their behavior. Government sectors or relevant organizations have to consider attitudes based on health motivation, perceived behavior control, and attitudes based on severity as the main issues affecting riding behavior, especially for women’s motorcycle riding behavior. Riders can be encouraged to have a behavior-influencing attitude through their health motivation by helping them to perceive the severity of motor vehicle accidents. Perceived behavior control could help riders to change their behavior. The greater the perception and realization of the danger of vehicular collisions, the safer the motorcycle riding behavior would become.

### 4.2. Discussion of LC

Multi-group SEM analysis using the locus of control (LC) showed that both internality and externality were behavior-influencing factors [31], in accordance with Rotter [12]. Factor analysis also supported behavioral-leading attitudes, in agreement with Champahom et al. [23], Lajunen and Räsänen [24], and Totkova [25]. The indicator other people [35], family or friend refer to Externality (EX) significantly following Transport Scotland [36] and Gicquel et al. [37], while accident news, public campaign on safe riding have also been involved in reducing unsafe driving behavior [38], as well as policy and law enforcement [39].

We found that internality (IN) influenced both men’s and women’s motorcycle riding behaviors. The effect was higher in women. Externality (EX) produced an influence similar to IN, where women were more highly influenced [16], which is a finding that agrees with that reported by Champahom et al. [23]. Considering IN, both men and women had a stronger influence than with EX. This result agrees with Champahom et al.’s [23] findings, which reported that IN had a stronger influence compared with EX and Arthur and Doverspike [19], that finding driving internality was related to accident rates rather than externality.

In terms of the conceptual and behavior-influencing attitudes based on sex classification, we found a significant difference in behavior. Therefore, policies should be constructed that can respond in different ways to the problem solving, support, and suggestions for each group according to context as attitudes and behaviors have different effects for men and for women.

## 5. Limitations and Future Work

The research limitations are that data from teenage self-reported riding behavior groups under 20 years of age were not collected, and we considered only the sex differences groups.

The research can be further developed by considering the indicators that most strongly affect the behavior of motorcycle riding in more detail, providing specific suggestions for safe riding policy specifications, helping public officials to reduce, avoid, and stop risky riding behavior, and helping the media to educate riders regarding the importance of riding safely.

## Figures and Tables

**Table 1:** Sample profile (*n* = 1516).

Variables	Men (*n* = 903)		Women (*n* = 613)	
	Frequency	Percent	Frequency	Percent
Age	Average age = 36.4 years		Average age = 33.2 years	
	SD = 9.57		SD = 9.71	
	Max = 72 years		Max = 70 years	
	Min = 20 years		Min = 20 years	
Average income	23,964 baht/month		21,721 baht/month	
Education level				
Other	1	0.11	-	-
Primary school	67	7.42	47	7.67
Junior high school	107	11.85	64	10.44
Senior high school	146	16.17	80	13.05
High vocational certificate	118	13.07	50	8.16
Bachelor’s degree	428	47.40	336	54.81
Master’s degree	22	2.44	34	5.55
Ph.D.	14	1.55	2	0.33
Occupation				
Student	57	6.31	71	11.58
Civil servant/state enterprise employee	39	4.32	21	3.43
Private companies	363	40.20	287	46.82
Personal business/trading owner	169	18.72	106	17.29
Agriculturist	79	8.75	19	3.10
Contractors	193	21.37	92	15.01
Housewife	-	-	17	2.77
Other	3	0.33	-	-
Licensed rider				
Yes	857	94.91	528	86.13
No	46	5.09	85	18.87

SD, standard deviation.

**Table 2:** Descriptive statistics.

Code	Latent Variable/Questionnaire	Men (*n* = 903)				Women (*n* = 613)			
		Mean	SD	Sk	Ku	Mean	SD	Sk	Ku
	Motorcycle Rider Behavior Questionnaire (MRBQ)								
TE	Traffic Error	1.88	0.56	−0.194	−1.250	1.82	0.54	−0.121	−1.303
CE	Control Error	2.33	0.51	−0.805	0.881	2.21	0.54	−0.740	0.394
ST	Stunt frequency	1.38	0.58	1.490	1.217	1.25	0.48	2.081	3.575
SE	Safety Equipment	1.82	0.88	1.158	1.172	1.71	0.79	1.241	1.294
	Theory of planned behavior (TPB) (X1–X19) and locus of control (LC) (X12–X20)								
X1	Road accidents caused by vehicle riding are the most dangerous ones.	6.60	0.63	−1.581	2.268	6.62	0.60	−1.588	2.512
X2	Health and the physical body are the most important factors when riding vehicles.	6.43	0.75	−1.027	0.046	6.46	0.71	−1.033	0.112
X3	Proper rest is the most important thing for vehicle riding.	6.22	0.74	−0.597	−0.277	6.33	0.71	−0.937	1.182
X4	You pay attention to safety when riding a vehicle.	6.29	0.74	−0.677	−0.353	6.41	0.74	−1.033	0.374
X5	If you get into an accident, your health and body will not be the same.	6.27	0.83	−0.952	0.288	6.29	0.91	−1.168	0.618
X6	If you do not wear a helmet, you may die if you get into an accident.	4.68	1.17	0.303	0.193	4.98	1.31	0.007	−0.465
X7	If an accident is caused by riding, it may cause death or disability, which require long-term treatment.	5.33	0.93	0.206	−0.347	5.55	0.98	0.090	−0.908
X8	Vehicle accidents would highly affect your study/work.	5.34	0.87	0.196	−0.140	5.53	0.95	0.136	−0.826
X9	Accidents would affect your life and network, e.g., immediate family, friends, relatives, etc.	5.37	0.90	0.269	−0.618	5.61	0.97	−0.013	−0.940
X10	Each accident causes death, mental illness, and loss of time and money.	5.50	0.95	−0.018	−0.700	5.69	0.98	–0.239	−0.743
X11	If you violate traffic laws, you may get fined or penalized.	5.56	0.97	−0.014	−0.463	5.73	1.06	–0.898	1.898
X12	Your family and friends drive carefully and follow traffic laws, so you do as well.	5.62	1.01	−0.352	−0.319	5.70	1.10	–0.689	−0.028
X13	You stay abreast of accident news, so you are afraid of accidents happening to yourself or your friends/family.	5.92	0.97	–0.565	−0.343	5.89	1.02	−0.548	−0.577
X14	You often see campaigns/public relations on safe riding.	5.92	0.99	−0.943	0.920	5.89	1.11	−0.925	0.401
X15	Your organization/company pays attention to safe riding/has a safe riding campaign.	5.59	1.18	−1.235	1.982	5.58	1.25	−1.091	1.287
X16	You make your own decisions to follow traffic laws independent of others.	6.25	0.84	−0.780	−0.419	6.22	0.80	−0.594	−0.690
X17	Helmet wearing is your own choice.	6.47	0.68	−0.993	0.103	6.46	0.66	−1.237	2.357
X18	Accidents are mostly caused by road conditions and the environment, not humans.	6.15	0.83	−0.666	0.018	6.24	0.78	−0.669	−0.333
X19	You can reduce the risk of accidents by riding safely.	6.15	0.81	−0.514	−0.562	6.26	0.73	−0.606	−0.373
X20	You find that polices are strict with regards to traffic discipline, so you pay attention to safe riding.	5.58	1.15	−0.323	−0.268	5.62	1.19	−0.435	−0.598

Note: X1–X20, code for TPB and LC indicators; SD, standard deviation; Sk, Skewness; and Ku, Kurtosis.

**Table 3:** Factor analysis for Men. *N* = 903, KMO for TPB = 0.778, and KMO for LC = 0.723.

Variable/Measurement Model/Cronbach’s α	EFA		CFA					
	Communalities	Loading	Loading	Est./S.E.	*p*-Value	Error Variance	CR	AVE
Motorcycle Rider Behavior Questionnaire (MRBQ)								
Traffic Error (TE)	-	-	0.843	43.557	<0.001	0.289	0.688	0.588
Control Error (CE)	-	-	0.624	26.840	<0.001	0.611		
Stunt (ST)	-	-	0.460	16.302	<0.001	0.788		
Safety Equipment (SE)	-	-	0.423	14.084	<0.001	0.821		
Attitudes based on Health Motivation (AHM) (Cronbach’s α = 0.806)								
X1	0.624	0.760	0.618	26.294	<0.001	0.618	0.812	0.676
X2	0.643	0.710	0.510	19.839	<0.001	0.740		
X3	0.558	0.664	0.692	34.446	<0.001	0.521		
X4	0.741	0.828	0.873	62.415	<0.001	0.237		
X5	0.615	0.701	0.687	34.524	<0.001	0.528		
Attitudes based on Severity (ASE) (Cronbach’s α = 0.927)								
X6	0.744	0.804	0.838	79.116	<0.001	0.298	0.930	0.827
X7	0.722	0.841	0.793	59.012	<0.001	0.371		
X8	0.759	0.830	0.902	86.089	<0.001	0.186		
X9	0.779	0.850	0.906	98.840	<0.001	0.178		
X10	0.843	0.900	0.874	89.500	<0.001	0.237		
X11	0.672	0.802	0.649	32.806	<0.001	0.578		
Subjective Norm (SN) (Cronbach’s α = 0.856)								
X12	0.643	0.791	0.640	36.335	<0.001	0.590	0.883	0.803
X13	0.702	0.769	0.981	111.048	<0.001	0.039		
X14	0.751	0.829	0.797	95.630	<0.001	0.365		
X15	0.685	0.816	0.793	59.077	<0.001	0.371		
Perceived Behavior Control (PC) (Cronbach’s α = 0.864)								
X16	0.756	0.856	0.674	34.408	<0.001	0.545	0.862	0.775
X17	0.614	0.769	0.624	25.119	<0.001	0.611		
X18	0.755	0.854	0.862	71.956	<0.001	0.257		
X19	0.744	0.827	0.938	90.679	<0.001	0.120		
Motorcycle Rider Behavior Questionnaire (MRBQ)								
TE	-	-	0.884	27.720	<0.001	0.219	0.697	0.595
CE	-	-	0.588	20.720	<0.001	0.654		
ST	-	-	0.449	14.149	<0.001	0.798		
SE	-	-	0.458	11.864	<0.001	0.790		
Externality (EX) (Cronbach’s α = 0.889)								
X12	0.560	0.746	0.621	31.755	<0.001	0.614	0.913	0.818
X13	0.716	0.840	0.962	69.801	<0.001	0.074		
X14	0.774	0.880	0.797	61.343	<0.001	0.364		
X15	0.712	0.843	0.871	71.119	<0.001	0.241		
X20	0.741	0.851	0.841	64.396	<0.001	0.293		
Internality (IN) (Cronbach’s α = 0.864)								
X16	0.744	0.853	0.671	33.708	<0.001	0.550	0.859	0.769
X17	0.603	0.768	0.598	23.304	<0.001	0.643		
X18	0.764	0.874	0.866	67.972	<0.001	0.250		
X19	0.751	0.866	0.942	81.001	<0.001	0.112		

Note: Extraction method: principal component analysis, Rotation method: Varimax with Kaiser Normalization. KMO, Kaiser–Meyer–Olkin; CFA, confirmatory factor analysis; CR, composite reliability; AVE, average variance extracted.

**Table 4:** Factor analysis for women. *N* = 613, KMO for TPB = 0.791, KMO for LC = 0.754.

Variable/Measurement Model/Cronbach’s α	EFA		CFA					
	Communalities	Loading	Loading	Est./S.E.	*p*-Value	Error Variance	CR	AVE
Motorcycle Rider Behavior Questionnaire (MRBQ)								
TE	-	-	0.817	37.225	<0.001	0.332	0.699	0.529
CE	-	-	0.787	32.988	<0.001	0.380		
ST	-	-	0.405	11.557	<0.001	0.836		
SE	-	-	0.360	10.865	<0.001	0.870		
Attitudes based on Health Motivation (AHM) (Cronbach’s α = 0.805)								
X1	0.605	0.741	0.683	25.21	<0.001	0.533	0.814	0.678
X2	0.615	0.736	0.489	14.726	<0.001	0.761		
X3	0.673	0.746	0.726	33.042	<0.001	0.473		
X4	0.731	0.824	0.839	47.331	<0.001	0.296		
X5	0.549	0.602	0.654	24.739	<0.001	0.573		
Attitudes based on Severity (ASE) (Cronbach’s α = 0.932)								
X6	0.780	0.796	0.865	79.142	<0.001	0.251	0.940	0.847
X7	0.702	0.807	0.797	52.144	<0.001	0.365		
X8	0.794	0.813	0.916	87.650	<0.001	0.161		
X9	0.832	0.881	0.932	113.294	<0.001	0.131		
X10	0.866	0.900	0.903	102.439	<0.001	0.184		
X11	0.652	0.794	0.666	31.065	<0.001	0.556		
Subjective Norm (SN) (Cronbach’s α = 0.900)								
X12	0.744	0.836	0.693	34.595	<0.001	0.520	0.917	0.852
X13	0.738	0.814	0.979	91.171	<0.001	0.042		
X14	0.807	0.872	0.832	63.628	<0.001	0.308		
X15	0.770	0.847	0.904	59.781	<0.001	0.183		
Perceived Behavior Control (PC) (Cronbach’s α = 0.842)								
X16	0.733	0.834	0.671	26.24	<0.001	0.549	0.847	0.756
X17	0.551	0.738	0.596	17.964	<0.001	0.645		
X18	0.767	0.865	0.857	50.731	<0.001	0.266		
X19	0.711	0.801	0.900	61.327	<0.001	0.190		
Motorcycle Rider Behavior Questionnaire (MRBQ)								
TE	-	-	0.945	25.861	<0.001	0.107	0.671	0.562
CE	-	-	0.651	20.518	<0.001	0.576		
ST	-	-	0.300	7.895	<0.001	0.91		
SE	-	-	0.350	7.506	<0.001	0.878		
Externality (EX) (Cronbach’s α = 0.921)								
X12	0.708	0.841	0.739	41.011	<0.001	0.453	0.936	0.860
X13	0.747	0.861	0.948	80.874	<0.001	0.101		
X14	0.813	0.901	0.857	76.796	<0.001	0.266		
X15	0.782	0.881	0.911	87.171	<0.001	0.169		
X20	0.780	0.875	0.845	61.173	<0.001	0.285		
Internality (IN) (Cronbach’s α= 0.842)								
X16	0.724	0.836	0.672	27.481	<0.001	0.548	0.843	0.751
X17	0.542	0.734	0.579	17.398	<0.001	0.665		
X18	0.769	0.875	0.857	49.589	<0.001	0.265		
X19	0.710	0.842	0.896	53.640	<0.001	0.197		

Note: Extraction method: principal component analysis, Rotation method: Varimax with Kaiser normalization.

**Table 5:** Correlation analysis results.

**Code (Men)**	**TE**	**CE**	**ST**	**SE**	**AHM**	**ASE**	**SN**	**PC**	**EX**	**IN**
TE	1.00									
CE	0.508 **	1.00								
ST	0.401 **	0.396 **	1.00							
SE	0.336 **	0.259 **	0.505 **	1.00						
AHM	−0.341 **	−0.351 **	−0.295 **	−0.334 **	1.00					
ASE	−0.323 **	−0.340 **	−0.132 **	−0.017	0.224 **	1.00				
SN	0.061	−0.191 **	0.051	−0.049	0.224 **	0.388 **	1.00			
PC	−0.479 **	−0.356 **	−0.208 **	−0.287 **	0.312 **	0.196 **	0.104 **	1.00		
EX	0.055	−0.205 **	0.032	−0.066*	0.245 **	0.414 **	0.986 **	0.128 **	1.00	
IN	−0.479 **	−0.356 **	−0.208 **	−0.287 **	0.312 **	0.196 **	0.104 **	1.000 **	0.128 **	1.00
**Code (Women)**	**TE**	**CE**	**ST**	**SE**	**AHM**	**ASE**	**SN**	**PC**	**EX**	**IN**
TE	1.00									
CE	0.592 **	1.00								
ST	0.291 **	0.318 **	1.00							
SE	0.224 **	0.239 **	0.403 **	1.00						
AHM	−0.387 **	−0.350 **	−0.292 **	−0.385 **	1.00					
ASE	−0.384 **	−0.477 **	−0.269 **	−0.099*	0.366 **	1.00				
SN	0.009	−0.287 **	−0.009	−0.122 **	0.240 **	0.467 **	1.00			
PC	−0.550 **	−0.371 **	−0.161 **	−0.172 **	0.295 **	0.182 **	0.046	1.00		
EX	0.000	−0.303 **	−0.025	−0.116 **	0.258 **	0.505 **	0.990 **	0.072	1.00	
IN	−0.550 **	−0.371 **	−0.161 **	−0.172 **	0.295 **	0.182 **	0.046	1.000 **	0.072	1.00

Note: ** *p*-value < 0.05.

**Table 6:** Model of fit and statistical and multi-group analyses.

Theory of Planned Behavior (TPB)	χ^2^	df	χ^2^/df	RMSEA	CFI	TLI	SRMR	Delta-χ^2^	Delta-df	*p*
Goodness-of-fit			<5	<0.08	>0.8	>0.7	<0.08			
Theory of planned behavior (TPB)										
Model 1: Men (n = 903)	766.104	163	4.70	0.064 (0.059–0.069)	0.957	0.933	0.065			
Model 2: Women (n = 613)	699.652	147	4.76	0.078 (0.073–0.084)	0.947	0.909	0.066			
TPB Measurement Invariance										
Model 3: Simultaneous	1420.039	303	4.69	0.070 (0.066–0.073)	0.954	0.924	0.068			
Model 4: Factor loading, intercept, and structural path held equal groups	1529.940	336	4.55	0.068 (0.065–0.072)	0.951	0.926	0.077	109.901	33	0.0000
Locus of Control (LC)										
Model 5: Men (n = 903)	92.597	31	2.99	0.047 (0.036–0.058)	0.991	0.976	0.038			
Model 6: Women (n = 613)	90.664	31	2.92	0.056 (0.043–0.070)	0.987	0.969	0.037			
LC Measurement invariance										
Model 7: Simultaneous	213.265	67	3.18	0.054 (0.046–0.062)	0.987	0.970	0.042			
Model 8: Factor loading, intercept, and structural path held equal groups	277.877	84	3.31	0.055 (0.048–0.062)	0.983	0.968	0.056	64.612	17	0.0000

The root mean squared error of approximation (RMSEA), standardized root mean square residual (SRMR), comparative fit index (CFI), and the Tucker–Lewis index (TLI).

**Table 7:** Theory of planned measurement model parameters.

Variable	Men					Women				
	Standardized Estimate	S.E.	Est./S.E.	*p*-Value	*R* ^2^	Standardized Estimate	S.E.	Est./S.E.	*p*-Value	*R* ^2^
Rider behavior use MRBQ by										
TE	0.841	0.019	43.260	<0.001	0.708	0.814	0.022	36.876	<0.001	0.663
CE	0.621	0.023	26.990	<0.001	0.386	0.785	0.024	32.918	<0.001	0.617
ST	0.458	0.028	16.307	<0.001	0.210	0.402	0.035	11.529	<0.001	0.162
SE	0.418	0.030	14.028	<0.001	0.175	0.363	0.033	11.028	<0.001	0.132
Attitudes based on Health Motivation (AHM) by										
X1	0.618	0.023	26.295	<0.001	0.382	0.684	0.027	25.277	<0.001	0.468
X2	0.508	0.026	19.751	<0.001	0.258	0.488	0.033	14.672	<0.001	0.239
X3	0.688	0.020	34.404	<0.001	0.473	0.733	0.021	34.521	<0.001	0.537
X4	0.870	0.014	61.765	<0.001	0.756	0.840	0.018	47.879	<0.001	0.705
X5	0.682	0.020	34.264	<0.001	0.466	0.665	0.025	26.862	<0.001	0.442
Attitudes based on Severity (ASE) by										
X6	0.838	0.011	79.202	<0.001	0.703	0.865	0.011	79.094	<0.001	0.748
X7	0.794	0.013	59.357	<0.001	0.631	0.797	0.015	52.117	<0.001	0.635
X8	0.902	0.010	86.630	<0.001	0.814	0.916	0.010	87.518	<0.001	0.839
X9	0.904	0.009	97.035	<0.001	0.818	0.932	0.009	112.354	<0.001	0.868
X10	0.871	0.010	88.353	<0.001	0.759	0.903	0.008	101.576	<0.001	0.815
X11	0.649	0.020	32.868	<0.001	0.422	0.665	0.022	30.911	<0.001	0.442
Subjective Norm (SN) by										
X12	0.636	0.018	36.069	<0.001	0.405	0.696	0.020	35.504	<0.001	0.485
X13	0.980	0.009	109.981	<0.001	0.960	0.980	0.011	92.916	<0.001	0.961
X14	0.794	0.008	96.966	<0.001	0.631	0.834	0.013	64.417	<0.001	0.695
X15	0.792	0.013	58.866	<0.001	0.628	0.906	0.015	61.312	<0.001	0.821
Perceived Behavior Control (PC) by										
X16	0.672	0.020	34.183	<0.001	0.451	0.669	0.026	26.019	<0.001	0.447
X17	0.617	0.025	24.818	<0.001	0.381	0.594	0.033	17.914	<0.001	0.352
X18	0.862	0.012	71.733	<0.001	0.744	0.859	0.017	51.543	<0.001	0.738
X19	0.936	0.010	89.163	<0.001	0.876	0.897	0.020	61.319	<0.001	0.805

**Table 8:** Locus of control measurement model parameters.

Variable	Men					Women				
	Standardized Estimate	S.E.	Est./S.E.	*p*-Value	*R* ^2^	Standardized Estimate	S.E.	Est./S.E.	*p*-Value	*R^2^*
Rider behavior use MRBQ by										
TE	0.884	0.032	27.719	<0.001	0.781	0.945	0.037	25.862	<0.001	0.893
CE	0.588	0.028	20.720	<0.001	0.346	0.561	0.032	20.518	<0.001	0.424
ST	0.449	0.032	14.149	<0.001	0.202	0.300	0.038	7.895	<0.001	0.090
SE	0.458	0.039	11.864	<0.001	0.210	0.350	0.047	7.506	<0.001	0.122
Externality (EX) by										
X12	0.621	0.020	31.756	<0.001	0.386	0.739	0.018	41.011	<0.001	0.547
X13	0.962	0.014	69.801	<0.001	0.926	0.948	0.012	80.874	<0.001	0.899
X14	0.797	0.013	61.343	<0.001	0.636	0.857	0.011	76.796	<0.001	0.734
X15	0.871	0.012	71.118	<0.001	0.759	0.911	0.010	87.172	<0.001	0.831
X20	0.841	0.013	64.395	<0.001	0.707	0.845	0.014	61.173	<0.001	0.715
Internality (IN) by										
X16	0.671	0.020	33.708	<0.001	0.450	0.672	0.024	27.481	<0.001	0.452
X17	0.598	0.026	23.304	<0.001	0.357	0.579	0.033	17.398	<0.001	0.335
X18	0.866	0.013	67.971	<0.001	0.750	0.857	0.017	49.589	<0.001	0.735
X19	0.942	0.012	81.001	<0.001	0.888	0.896	0.017	53.640	<0.001	0.803

**Table 9:** Results of the hypothesis testing.

Hypothesis	Men			Women		
	Standardized Estimates	*t*-Value	Result	Standardized Estimates	*t*-Value	Result
Direct effect						
Theory of planned (TPB)						
H3: PC → MRB	−0.411	−13.825 **	Supported	−0.323	−7.744 **	Supported
H4: AHM → MRB	−0.458	−11.732 **	Supported	−0.751	−9.360 **	Supported
H5: ASE → MRB	−0.215	−5.835 **	Supported	−0.168	−3.023 **	Supported
H6: SN → MRB	0.000	−0.012	-	−0.025	−0.690	-
Locus of control (LC)						
H7: EX → MRB	−0.227	−5.169 **	Supported	−0.382	−7.245 **	Supported
H8: IN → MRB	−0.586	−19.342 **	Supported	−0.607	−7.015 **	Supported
Indirect effect						
AHM → PC	0.310	9.613 **	-	0.288	7.027 **	-
ASE → AHM	0.320	10.129 **	-	0.411	11.492 **	-
SN → ASE	0.418	15.494 **	-	0.455	14.891 **	-

Note: ** *p*-value < 0.05.
